# Grado de fiabilidad de la evaluación del análisis de Bolton en modelos virtuales tridimensionales versus modelos de yeso. una revisión

**DOI:** 10.21142/2523-2754-1102-2023-155

**Published:** 2023-06-29

**Authors:** Humberto Loma Salcedo, Nelly Erlinda Huasco Huarcaya

**Affiliations:** División de Ortodoncia, Universidad Científica del Sur. Lima, Perú. humbertoloma.s@hotmail.com , lozanoh77@hotmail.com Universidad Científica del Sur División de Ortodoncia Universidad Científica del Sur Lima Peru humbertoloma.s@hotmail.com lozanoh77@hotmail.com

**Keywords:** modelos virtuales, modelos de yeso, Bolton, fiabilidad, virtual models, plaster models, Bolton, reliability

## Abstract

**Introducción:**

El análisis de Bolton se emplea para determinar anomalías con respecto a la masa dentaria, con fines de diagnóstico y planificación del tratamiento, la posibilidad de utilizar un método digital, que fue introducido y probado para medir el tamaño del diente mesiodistal, es una alternativa atractiva; además, la creciente aceptación de la tecnología dental digital, generó que los modelos de estudio digitales se vuelvan populares en entorno a la ortodoncia.

**Objetivo:**

evaluar el grado de fiabilidad de la evaluación del análisis de Bolton en modelos virtuales tridimensionales versus modelos de yeso por medio de una revisión de la literatura.

**Materiales y métodos:**

Se realizó una búsqueda en las principales bases de datos de la literatura científica internacional sobre ciencias de la salud: Medline, a través de PubMed, SciELO, Lilacs, Embase. Se incluyeron artículos de revisión publicados entre el año 2000 hasta octubre 2021.

**Resultados:**

El escáner de sobremesa fue la mejor opción para la digitalización de modelos dentales, pero esto no quita mérito alguno al CBCT y al escáner láser intraoral, que siguen siendo una opción asequible válida para la digitalización de modelos 3D con resultados que se encuentran dentro del rango “clínicamente aceptable”.

**Conclusiones:**

Los modelos digitales 3D generados con escáneres intraorales y escáneres extraorales son confiables y precisos en comparación con las impresiones convencionales y ahorran tiempo. En la mayoría de los casos, se hallaron diferencias estadísticamente significativas, pero de poca relevancia clínica.

## Abstract (en)

**Introduction:**

Bolton analysis is used to determine anomalies with respect to the dental mass, for diagnosis and treatment planning purposes, the possibility of using a digital method was introduced and tested to measure the size of the mesiodistal tooth. In this way, digital dental technology has made digital study models become popular in orthodontics.

**Objective:**

to evaluate the degree of reliability of the evaluation of the Bolton analysis in three-dimensional virtual models versus plaster models through a review of the literature.

**Materials and methods:**

A search was carried out in the primary databases of the international scientific literature on health sciences: Medline, through PubMed, SciELO, Lilacs, and Embase. Review articles published between the year 2000 and October 2021 were included.

**Results:**

The desktop scanner was the best option for digitizing dental models, but this does not detract from CBCT and intraoral laser scanners, which are still an option. Reasonable validity for digitizing 3D models with results within the "clinically acceptable" range was found.

**Conclusions:**

3D digital models generated with intraoral and extraoral scanners are reliable and accurate compared to conventional impressions and save time, in most cases statistically significant differences were found, but of little clinical relevance.

## INTRODUCCIÓN

Los modelos de estudio son una herramienta importante en el diagnóstico y planificación del tratamiento de ortodoncia, los mismos que documentan los estados iniciales de maloclusión, proceso y resultado del tratamiento en las tres dimensiones. Estos, en ocasiones, son asociados con problemas como el almacenamiento, fracturas y pérdidas ^1^^-^^3^. El análisis de Bolton se emplea para determinar anomalías con respecto a la masa dentaria, con fines de diagnóstico y planificación del tratamiento. En1958, Bolton evaluó pacientes con oclusiones ideales y estableció dos proporciones utilizando las sumas de los anchos mesiodistales de los dientes maxilares y mandibulares. Si bien es cierto que el análisis de Bolton permite al clínico determinar la discrepancia de la masa dentaria y el grado de diferencia con la proporción ideal; los modelos dentales de yeso pueden romperse, fracturarse y tener degradación ^4^^,^^5^. Tradicionalmente, los índices de Bolton se realizaban manualmente, por lo que la posibilidad de utilizar un método digital, que fue introducido y probado para medir el tamaño del diente mesiodistal, es una alternativa atractiva; además la creciente aceptación de la tecnología dental digital generó que los modelos de estudio digitales se vuelvan populares en el área de la ortodoncia ^6^^-^^9^. La capacidad de la tecnología de modelos digitales está estimulando una adopción más rápida y amplia de esta tecnología en clínicas e instituciones de ortodoncia ^7^^,^^8^. Los almacenamientos de los modelos de yeso, por su tamaño y peso, implican esfuerzos considerables y requisitos de espacio en comparación con otra forma de documentar el tratamiento. Varios estudios han evaluado la precisión de los modelos digitales y estos han informado que parecen ser clínicamente aceptables y reproducibles para realizar diagnósticos de ortodoncia. Una de las ventajas de las impresiones digitales que utilizan un escáner intraoral es su eficacia en pacientes con fuertes reflejos al vómito; asimismo, es posible sobrescribir solo la zona donde la impresión no es clara ^9^^,^^10^. Hay varias técnicas disponibles para obtener modelos digitales 3D, como las obtenidas con tomografía computarizada de haz cónico (CBCT), escaneo láser intraoral (ILS) y los escáner extraorales, que muestran una alta correlación en su precisión ^(^^11^^-^^15^. De manera similar, algunos estudios corroboraron la confiabilidad de los modelos CBCT versus 2D para calcular el análisis de Bolton, los cuales encontraron ciertas diferencias poco significativas clínicamente. Otra ventaja de los modelos digitales es que, a través de ellas, se puede intercambiar diagnósticos y planes de tratamiento con otros especialistas, acortando el tiempo y la distancia. Dicho esto, los modelos de estudio digital hoy en día son una alternativa muy atractiva en comparación a los modelos de yeso ^4^^,^^15^^-^^18^. 

Al ser comparadas las medidas mesiodistales de las piezas dentarias de los modelos virtuales versus los modelos de yeso (estándar de oro) y probar la correspondencia de las medidas, se contribuirá de manera confiable con los conocimientos científicos en el área de la ortodoncia y otras especialidades odontológicas. Para que su uso sea más confiable y rutinario de los modelos 3D en el área de ortodoncia, es necesario saber si las medidas obtenidas en el ordenador son compatibles a las medidas realizadas manualmente en los modelos de yeso. Además, se debe saber si los programas disponibles que generan imágenes 3D son fiables para realizar el análisis de Bolton, así como el uso de diferentes *softwares* presenten el mismo nivel de precisión. En diversos países del mundo, la digitalización en ortodoncia no es ajena y con la llegada de los modelos digitales 3D se ha cambiado la forma tradicional de diagnosticar en las mediciones manuales de los modelos de estudio, lo que a su vez permite eliminar todas las desventajas que estas representan. Es por ello que se considera de gran impacto e importancia profundizar el estudio del uso y aplicación de los análisis en los modelos de estudio digitalizados, lo que permitirá, en un futuro no muy lejano, la determinación de un gran número de análisis de una forma rápida, sencilla y exacta. En este sentido, el propósito de esta revisión de literatura fue determinar el grado de fiabilidad del análisis de Bolton en modelos virtuales tridimensionales versus modelos de yeso (*gold standard*) a través de una revisión de la literatura científica.

## MATERIALES Y MÉTODOS

### Protocolo y registro

Está investigación fue registrada en el comité de ética e investigación de la Universidad Científica del Sur con el número de registro 6761-1. La presente revisión de la literatura utilizó el protocolo PRISMA, para lo cual se planteó la siguiente pregunta PECOS de investigación:

(P): Ancho mesiodistal de las piezas dentarias para análisis de Bolton en individuos completamente dentados hasta segundos molares. 

(E): Modelos virtuales tridimensionales mediante el uso de la tecnología 3D

(C): Modelos de yeso convencionales

(O): Fiabilidad de la evaluación del análisis de Bolton en modelos virtuales tridimensionales

(S): Estudios longitudinales, observacionales, y estudios de cohortes

### Estrategia de búsqueda

Dos investigadores capacitados y calibrados realizaron una búsqueda bibliográfica en las principales bases de datos de la literatura científica internacional sobre ciencias de la salud: Medline, a través de PubMed, SciELO, Lilacs y Embase, utilizando las palabras claves: modelos virtuales, modelos de yeso, Bolton, fiabilidad. Se incluyeron artículos de revisión publicados entre el año 2000 hasta octubre del 2021, y se procedió a su lectura, según los criterios de inclusión y exclusión 

### Términos de búsqueda

La estrategia de búsqueda utilizada fue la siguiente:

((((((((((RELIABILITY* ANALYSIS* DENTAL) OR (VIRTUAL MODELS*)) OR (PLASTER MODELS*)) AND (DENTAL BOLTON ANALYSIS*)) AND (RELIABILITY VIRTUAL MODELS*)) OR (RELIABILITY ANALYSIS DENTAL*)) AND (INTRAORAL DENTAL SCANNER*)) AND (EXTRAORAL DENTAL SCANNER*)) AND (3D DENTAL SCANNER*)) OR (3D DENTAL SCANNER RELIABILITY*)) OR ((((VIRTUAL* MODELS*) AND (PLASTER* MODELS*)) AND (DENTAL* BOLTON* ANALYSIS*)) AND (RELIABILITY*))

### Criterios de inclusión y exclusión

Las publicaciones incluidas fueron aquellas en idioma inglés y español que, en su contenido, abarcaran modelos virtuales tridimensionales en odontología, fiabilidad de modelos virtuales tridimensionales dental, usos de distintos tipos de tecnología para generar modelos virtuales tridimensionales en odontología, fueron excluidas las publicaciones que no se encontraban dentro del rango 2000 hasta octubre de 2021, no portaban el criterio de diagnóstico o no reportaban resultados con relación a modelos virtuales tridimensionales en el análisis de Bolton (Tabla 1).

**Tabla 1:** Número de estudios excluidos con razones después del texto completo

Razones de exclusión	(n)
Elegibilidad	7
No reporta criterio de diagnóstico	2
No reporta resultados 3D	1
No reporta resultados Bolton	6
Suma	16

### Proceso de selección

Se realizó la búsqueda en PubMed, SciELO, Lilacs y Embase, con términos Mesh y la antigüedad establecida en los criterios de inclusión. Se identificaron 85 artículos relacionados con modelos virtuales en odontología y su fiabilidad de su uso para realizar el análisis de Bolton. Se revisaron los resúmenes de los artículos y se descartaron los artículos que no cumplían con los criterios de selección, por lo que se obtuvo un total de 21 artículos científicos para ser analizados minuciosamente. 

## RESULTADOS

### Selección de estudios

**Figura 1:**
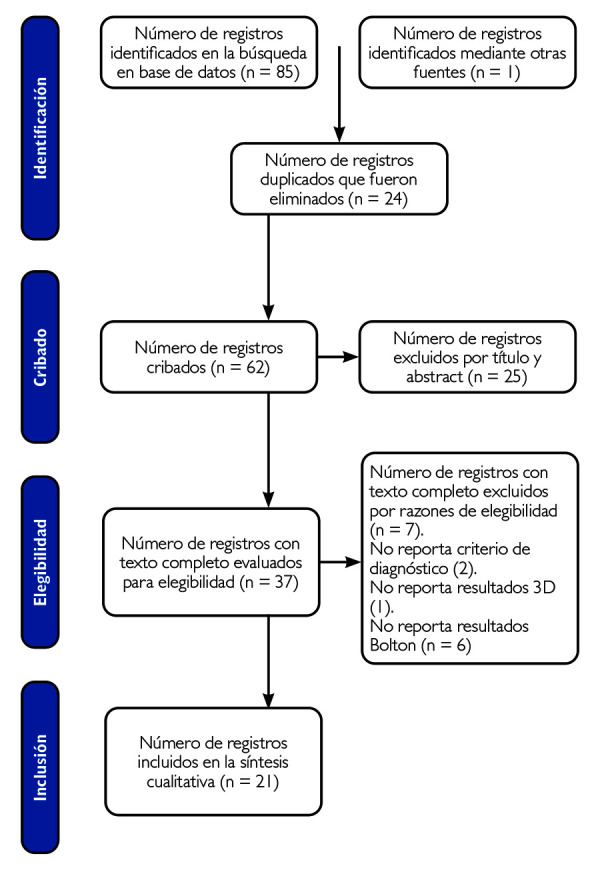
Diagrama de flujo del proceso de selección de los estudios

De la búsqueda en las bases electrónicas y las referencias de los estudios, se recuperó un total de 85 publicaciones y de las referencias de otros estudios se identificó un artículo. Tras la eliminación de referencias duplicadas y la evaluación manual del título y resumen se obtuvieron 37 artículos para la lectura completa. Después de la lectura, evaluación de la calidad metodológica y discusión, 16 estudios se excluyeron debido a elegibilidad, porque no portaban el criterio de diagnóstico o no reportaban resultados con relación a modelos virtuales tridimensionales en el análisis de Bolton, lo que derivó en un total de 21 artículos considerados elegibles para la síntesis cualitativa (Fig. 1).

## DEFINICIÓN DE ANÁLISIS DE BOLTON Y TÉCNICAS DISPONIBLES PARA GENERAR MODELOS VIRTUALES TRIDIMENSIONALES

### Análisis de Bolton

El análisis de Bolton utiliza el ancho mesiodistal del diente para calcular una suma de las relaciones entre el ancho del diente mandibular y el maxilar necesarias para una oclusión adecuada, compara la proporción que existe en la sumatoria de los anchos mesiodistales de doce dientes mandibulares, que es de la pieza 36 a la 46, versus la sumatoria de sus homólogos superiores, que es de 16 a 26, a lo cual se le conoce como Bolton 12 o Bolton total. Por otro lado, también compara la sumatoria de los seis dientes anteriores inferiores, que es de 33 a 43, versus la sumatoria de sus homólogos superiores, que es de 13 a 23, tomando el nombre de Bolton 6. La utilización de este índice detecta posibles desarmonías antes del comienzo del tratamiento, esto con respecto a discrepancia de masa dentaria. Dicho esto, el índice de Bolton anticipa las alteraciones de masa dentaria que se pueden observar al finalizar el tratamiento de ortodoncia ^19^^-^^23^.

### Discrepancia de Bolton 6 y Bolton 12

El análisis de Bolton es empleado con propósitos de prever ciertas discrepancias de masa dentaria con fines de diagnóstico y plan de tratamiento. Bolton analizó en pacientes con una oclusión ideal y estableció dos radios usando la suma de los anchos mesiodistales de los dientes superiores e inferiores; esto permite detectar las discrepancias de dicha masa dentaria y el grado de diferencia del radio ideal. El índice de Bolton 12 en un paciente ideal debe mostrar un valor dentro de la norma que es de 91,3, con desviación estándar ± 1,91; si la relación total excede de 93,21, la discrepancia es por exceso de masa dentaria inferior; si la relación es menor que 89,39, la discrepancia es por un exceso de masa dentaria superior. Lo mismo se realiza para los seis dientes anteriores, Bolton 6, el cual debe mostrar un valor dentro de la norma que es de 77,2 con una desviación estándar ±1,65; si la proporción anterior supera 78,85, indica un exceso de material dentario inferior; valores inferiores a 75,55, un exceso de masa dentaria superior. Se recomienda el uso de tablas o fichas para su mejor aplicación.

### Técnicas disponibles para generar modelos virtuales tridimensionales

Existen varias técnicas disponibles para obtener modelos digitales 3D, desde CBCT, el escaneo láser intraoral (ILS) o el escaneo extraoral de los modelos de yeso. A partir de la introducción de los modelos digitales, varios estudios han sido llevados a cabo con la finalidad de evaluar la fiabilidad de las medidas obtenidas en un modelo. Se debe recordar que las unidades de tomografía se clasifican según su volumen de la imagen o campo de visión, “field of view” (FOV). A mayor FOV se obtienen imágenes más extensas; sin embargo, estas son de menor resolución en relación con un sistema de FOV limitado, que permite obtener una imagen de mayor resolución, pero solo de una parte pequeña de la cara. Para la aplicación en ortodoncia se debe seleccionar un CBCT que brinde una mejor resolución ^11^^,^^20^^-^^25^.

El escaneo láser intraoral (ILS) genera modelos dentales 3D de alta precisión e información de diagnóstico, sin la necesidad de tomar impresiones, pero no brinda información esquelética adicional. Estos son dispositivos de fácil manipulación, ligeros y ergonómicos; en el mercado se encuentran de diferentes tamaños y modelos con puntas desmontables y autoclavables ^11^^,^^24^^-^^26^. 

### Escáneres extraorales

Un escáner extraoral reproduce digitalmente un modelo ya tomado en el paciente el cual, a su vez, va a reproducir en detalle el medio intrabucal del paciente, con todas las ventajas que esto conlleva ^26^^,^^27^. Hoy existen muchos escáneres extraorales disponibles; sin embargo, solo algunos son para su uso en ortodoncia.

### Serie 3Shape R

3Shape produce una infinidad de modelos y escáneres de impresión para las diferentes especialidades odontológicas. Su escáner de la serie R500TM utiliza una tecnología de escaneo láser y dos cámaras de 1,3 megapíxeles para crear modelos de estudio digitales indirectos a partir de modelos de estudio e impresiones. Sin embargo, la serie R700TM está diseñada para laboratorios más grandes, a los que les toma menos tiempo de escaneo de modelos e impresiones. Por último, el escáner de la serie R900TM es capaz de escanear a color, con una reducción en el tiempo y además brinda una mayor precisión a través de la tecnología de la luz LED azul y cámaras de 45.0 megapíxeles^27^.

### Sistema de escaneo de escritorio Ortho Insight 3D

Este escáner de mesa utiliza brazos de escaneo para proporcionar tres ejes de movimiento; además, tiene un volumen máximo de escaneo de 46 462 pulgadas y una precisión de 40-200 micras, según la resolución seleccionada. Por otro lado, los escaneos se guardan en un formato de archivo abierto flexible que puede ser STL, OBJ o PLY. El paquete incluye el *software* Ortho Insight 3D y hay una opción para comprar módulos de *software* opcionales adicionales para la unión indirecta y la cefalometría de Ortho Insight ^28^^,^^29^.

### Sistemas de escaneo y diseño de Dental Wings

Dental Wings pone a disposición una gama de escáneres para uso en clínicas o laboratorios. Sus escáneres 7Series e iSeries escanean modelos de yeso e impresiones dentales, mientras que 3Series se limita a escanear solo modelos de yeso. Los fabricantes introducen un módulo como un “complemento” para su *software* DWOSTM, el cual permite diseñar modelos de estudio de ortodoncia para el archivo o fabricación de modelos digitales ^30^.

### CBCT

La tomografía ayuda a tener un buen diagnóstico y, por consiguiente, armar el plan de tratamiento de la mejor manera. Sin embargo, no todas las CBCT ofrecen la misma resolución; en el mercado existen diferentes tomógrafos. El vóxel, mínima unidad de un volumen de tomografía, está relacionado con la resolución de las imágenes; a menor vóxel mejor resolución de las imágenes. Las CBCT también ofrecen diferentes parámetros de campo de imagen o “*field of view*” (FOV). Algunas tomografías ofrecen campo completo, otras permiten realizar tomografías de los tres campos, y otras se limitan a un campo mediano, y mientras más pequeño sea el campo de imagen va a dar una mayor resolución y esta característica no es propia de todos los tomógrafos, y recordar que a mayor resolución menor cantidad de vóxel. Es decir, tomógrafo de campo pequeño ofrecen vóxel pequeño y relación directa. Se toman dos puntos de referencia que coinciden con el ancho mesiodistal de los dientes, el cual se mide con un calibrador digital y, para minimizar los errores, se sugiere que un solo operador realice el proceso de análisis, lo que da como resultado 48 puntos de referencia por escaneado mediante CBCT de cada paciente. Para cada punto de referencia se graban coordenadas x, y, z, y los valores de las medidas del ancho mesiodistal de los dientes se obtienen calculando las distancias entre las coordenadas de los dos puntos de referencia correspondientes ^31^^,^^32^.

### Escáneres intraorales

Sirve esencialmente para obtener una imagen dental exacta de la cavidad bucal del paciente y transmitirlos al ordenador inmediatamente, con lo que se obtiene toda la información y ayuda necesaria para un diagnóstico rápido que beneficia en tiempo y costo tanto al paciente como al profesional. Este escáner está compuesto por tres partes principales: el *software*, la computadora y la cámara con la que se llevará a cabo el procedimiento. Funciona a través de un haz de luz que choca con los tejidos en la cavidad bucal y, en su retorno, captura la forma del objeto y la transmite al ordenador, y así se puede observar una imagen real del estado completo de la boca del paciente ^10^^,^^33^. Existen muchos escáneres intraorales disponibles, pero solo algunos se usan para ortodoncia.

### Escáner intraoral iTeroH, comercializado por Align Technology Inc.

El escáner iTeroH utiliza una tecnología de imagen confocal, con ayuda de rayos láser de luz roja. Los formatos de datos abiertos STL son compatibles con otros sistemas de *software* de restauración u ortodoncia. Utiliza una base de datos en la nube para el almacenamiento de los datos y ha sido diseñado para usarse con un *software* OrthoCadH e InvisalignH ClincheckH. Esto permite al ortodoncista verificar y ajustar el plan de tratamiento prescrito para una maloclusión al emplear la tecnología InvisalignH. Además, el *software* InvisalignH Result Simulator ha sido diseñado para simular los resultados del tratamiento con los alineadores InvisalignH ^30^.

### Escáner intraoral TRIOSH comercializado por 3Shape

Este escáner utiliza la tecnología Ultrafast Optical Sectioning. Los datos de superficie se crean uniendo muchas porciones de datos recibidos del escáner. Está disponible en dos formatos: TRIOS Cart, el cual es una unidad móvil independiente con *software* integrado de pantalla táctil, y TRIOS Pod, disponible para la conexión a un iPad, computadora portátil o monitor integrado en la unidad dental. El formato de archivo STL abierto es compatible con cualquier programa que admita dicho formato, además de tener conectividad inalámbrica y Bluetooth integrado. El escáner TRIOS se puede usar con el programa de *software* OrthoAnalyzerTM; sin embargo, este ha sido diseñado para facilitar el diagnóstico y la planificación del tratamiento con herramientas que ayudan a medir el *overjet*, la sobremordida, el apiñamiento, la longitud del arco y la forma ^30^.

### Escáner True Definition, comercializado por 3M ESPE

Este es un escáner intraoral muy ligero que emplea tecnología de video 3D con luz LED azul. Este escáner intraoral está conectado a un monitor de pantalla táctil en una unidad móvil que se puede transferir entre los sillones dentales. Este sistema requiere un medio de contraste, una ligera capa de polvo de plata, el mismo que está conectado a la unidad para facilitar su uso. Los datos se recopilan en un formato STL abierto y el almacenamiento se logra a través de la nube, en el 3MTM Connection Center. Los archivos de datos están diseñados para usarse con UnitekTM Software de modelo digital para herramientas de planificación del tratamiento, como el cálculo de la relación de análisis de Bolton y el análisis de espacio. Las unidades tienen wi fi habilitado ^30^^,^^34^.

### CS 3600 Scanner Intraoral Color

Esta tecnología utiliza una técnica de láser azul, captura la imagen digital en tiempo real, tiene la capacidad de pausar y recuperar datos en un momento posterior. El formato STL es abierto, los *softwares* disponibles pueden ser de una versión básica con el que se puede ver y realizar mediciones para la planificación del tratamiento y la versión avanzada permite configuraciones virtuales, segmentación dental, simulación de resultados de tratamientos, y se puede usar en conjunto con unidades de rayos X 3D. Su conectividad es compatible con cualquier computadora, solo que requiere la versión de 64 bits de Windows 7 Profesional ^27^^,^^34^.

### Digitalización de modelos

Se pueden digitalizar con escáneres de escritorio (escáner extraoral), escáneres intraorales y, recientemente, también con CBCT se obtienen modelos digitales en el ordenador. Existen dos métodos, el primero es la digitalización de los modelos de yeso u otro material de impresión, y el segundo es la digitalización directamente en la cavidad bucal del paciente. Desde el año 2001, se han comercializado varios sistemas para la evaluación digital; los modelos digitales directos requieren el uso de un escáner intraoral y el método indirecto requiere de un láser escáner o imágenes tomográficas computarizadas de impresiones o modelos de yeso, que luego son convertidos en imágenes digitales y guardados por los servidores de los fabricantes; de esta manera, quedan disponibles para ser descargados mediante la cuenta del titular. El fabricante provee el *software* para la realización de las medidas. Para ser lanzadas al mercado, estas herramientas fueron sometidas a pruebas de calidad en las que se tomaron medidas en relación con el análisis de Bolton que no presentaron diferencias significantes respecto del *gold standard* y fueron clínicamente aceptables ^25^^,^^35^^-^^37^.

En la década pasada, se ha visto la aparición de modelos digitales con una calidad aceptable, lo cual permite que las historias clínicas ortodónticas sean digitalizadas. Sin embargo, para los ortodoncistas, lo más importante de un modelo digital es la exactitud y la fiabilidad, sobre todo la comodidad de obtener datos reales en 3D y realizar sus diagnósticos basados en RX, fotografías y proyecciones que los modelos digitales ofrecen. Se debe tener en cuenta que, durante el proceso de escaneado, ciertos artefactos pueden afectar la calidad de la imagen; además, por el movimiento del paciente y una pobre reconstrucción de superficies oclusales. 

Otra desventaja es que los archivos de modelos digitales obtenidos mediante CBCT no vienen en el mismo formato o aspecto que los autores no especifican ^29^^,^^38^^-^^44^. Por ejemplo, para la digitalización 3D y transformación al formato STL con el programa Nemocast, primero se hace la orientación en los tres ejes x, y, z, para luego realizar la colocación de los planos oclusales y rafe medio, a fin de continuar con la colocación de los puntos mesiodistales en las piezas incluidas dentro del análisis, y el programa realiza el zocalado según la norma ABO. Ya con el modelo digitalizado, el programa proporciona los datos requeridos para el diagnóstico, que puede ser el índice de Bolton, la discrepancia de arco, entre otros.

### *Software* para análisis de modelos digitales para el análisis de Bolton

En la actualidad, el análisis de modelos digitales está ganando aceptación en ortodoncia, pero su fiabilidad depende de *hardwares* y *softwares* usados en la digitalización. El *software* para el análisis del modelo digital debe ofrecer resultados que sean confiables y válidos, como los del análisis de modelos de yeso. Sin embargo, existe *software* interno y externo; los primeros son aquellos que vienen junto con el producto al ser adquirido, mientras que los segundos se adquieren aparte. En caso de avería del *software* interno, existe una gama de *software* en el mercado. No obstante, diversos autores consideran que el modelo digital de OrthoCAD tiene la mejor legibilidad, un gran contraste entre colores y espacio entre elementos, pero es el OrthoAnalyzer el que presenta un sistema con mejores condiciones para medir el *overjet* y el *overbite*^45^^-^^49^.

## DISCUSIÓN

### Sumario de la evidencia

Debido al alto interés del uso de los dispositivos que generan imágenes 3D, como alternativa para minimizar el riesgo de almacenamiento, fracturas y pérdidas de los modelos de yeso durante el análisis de Bolton, se han realizado muchos estudios para determinar la fiabilidad de los escáneres 3D, tanto los extraorales como los intraorales. La introducción de los flujos de trabajo digitales en ortodoncia significa una mayor eficiencia y facilita el almacenamiento de datos, la reproductibilidad y la documentación del tratamiento, e incluso puede conducir a conceptos de tratamiento avanzados. La mayoría de los estudios incluidos tienen una baja a moderada calidad metodológica y bajo nivel de certeza para los resultados evaluados. La revisión técnica sugiere consistentemente que los escáneres generadores de modelos virtuales para el análisis de Bolton son efectivos, lo que permite un gran número de análisis de una forma rápida, sencilla y exacta. En resumen, los modelos digitales permiten intercambiar diagnósticos y planes de tratamiento con otros especialistas, acortando el tiempo y la distancia, por lo que, hoy en día, son una alternativa muy atractiva en comparación a los modelos de yeso ^4^^,^^15^^-^^18^. 

Existen muchos estudios que llegan a conclusiones basadas en sus propios diseños metodológicos. Aragón *et al*. ^17^ compararon la validez y confiabilidad de los escáneres intraorales versus modelos de yeso convencionales. Los escáneres evaluados fueron D250, Lava COS, Ortho Proof, iTero, iOC intraoral y Lava. Dicho estudio evaluó la confiabilidad de los anchos mesiodistales de los dientes para el análisis de Bolton. Las mediciones a partir de los modelos digitales intraorales parecieron ser fiables y precisas, en comparación con las impresiones convencionales. Por otro lado, Emara *et al*. ^50^ realizaron una evaluación comparativa de la digitalización del modelo de yeso dental haciendo uso de diferentes tecnologías de escaneo. El escáner extraoral se mostró como una mejor opción; sin embargo, el CBCT demostró ser una alternativa muy precisa. Si bien el escáner intraoral mostró resultados más bajos en términos de precisión, sigue siendo una opción válida, dado que presenta resultados que se encuentran dentro del rango “clínicamente aceptable”.

Camardella *et al*. ^23^ compararon la precisión y confiabilidad de las mediciones realizadas utilizando los programas diferentes de *software* Ortho Analyzer (3Shape) y Digimodel (OrthoProof). Se escanearon modelos de yeso con un escáner láser 3Shape y un escáner Flash CT y no se encontraron diferencias estadísticamente significativas entre las medidas del modelo de yeso versus el modelo digital. El *software* utilizado no influyó en la precisión de las mediciones. Por ello, tanto los métodos de fabricación como el *software* podrían usarse indistintamente.

San José *et al*. ^2^ compararon la fiabilidad y precisión de las mediciones dentales directas e indirectas derivadas de dos modelos 3D generados por escáner intraoral iTero (Algin Tecnology, Amsterdam, Países Bajos) y CBCT segmentado, que se obtuvieron utilizando el Planmeca Promax 3D (Planmeca, Helsinki, Finlandia), y se les comparó con un modelo 2D, escaneado con un escáner convencional. El error intraobservador e interobservador para el modelo del escáner intraoral fue inferior a 0,44 mm y para el CBCT segmentado, inferior a 0,97 mm, por lo que ambos se encontraron dentro de los límites de aceptación clínica.

Kim *et al*. ^4^ compararon la precisión del análisis de Bolton obtenido a partir de modelos digitales escaneados con escáner láser tridimensional (3D) Ortho Insight versus imágenes de tomografía computarizada de haz cónico y modelo de yeso. Las relaciones Bolton 6 y Bolton 12 obtenidas por las tres modalidades diferentes mostraron una excelente concordancia (>0,970). Asimismo, los resultados del coeficiente de correlación intraclase CCI fueron óptimos (>0,858 para las tres modalidades).

Finalmente, Rossini *et al*. ^26^, en una revisión sistemática cuyo objetivo fue evaluar la exactitud, validez y confiabilidad de las medidas obtenidas a partir de los modelos de yeso, hallaron que los modelos digitales son tan fiables como los de yeso, con una alta precisión, reproductibilidad y fiabilidad.

### Limitaciones y futuras direcciones

La extracción de los datos y el análisis cualitativo, generalmente realizado en descripciones cualitativas sistemáticas, presentan marcados inconvenientes en comparación con el uso del metaanálisis, por lo que la posibilidad de extrapolar resultados es limitada.

El presente trabajo proporciona una base de evidencia con estudios seleccionados de calidad metodológica, que puede ayudar a los odontólogos en la incorporación de los modelos digitales 3D en su práctica clínica. Esto disminuirá considerablemente el hacinamiento de los modelos de yeso y permitirá su almacenamiento sin ocupar espacio físico alguno. 

De acuerdo con varios estudios, existe sustento para tener en cuenta como una opción válida y fiable los modelos virtuales tridimensionales, ya que son una herramienta fiable y que contribuye al buen diagnóstico y la planificación de los tratamientos. Además, los resultados de la presente revisión de literatura contribuirán a futuras investigaciones.

## CONCLUSIONES

Los modelos digitales 3D generados con escáneres intraorales y extraorales parecen ser confiables y precisos en comparación con las impresiones convencionales, y ahorran tiempo de trabajo. Aunque en la mayoría de los artículos se hallaron diferencias estadísticamente significativas, clínicamente, las consideraron no relevantes, salvo para el diagnóstico dental utilizando diferentes tecnologías de escaneo, en donde el escáner de sobremesa parece ser la mejor opción de digitalización de modelos dentales por su precisión. Aun así, la CBCT y el escáner láser intraoral siguen siendo una opción válida para la digitalización de modelos 3D, con resultados que se encuentran dentro del rango “clínicamente aceptable”. 

## Fuente de financiamiento:

Autofinanciado
